# Transdiaphragmatic single-port video-assisted thoracoscopic surgery; a novel approach for pulmonary metastasectomy through laparotomy incision – case series

**DOI:** 10.1186/s13019-021-01393-2

**Published:** 2021-02-19

**Authors:** Riad Abdel Jalil, Mohamad K. Abou Chaar, Omar M. Shihadeh, Obada Al-Qudah, Azza Gharaibeh, Loulia Aldimashki, Ali Dabous, Rami Ghanem, Ahed Al-Edwan

**Affiliations:** 1 Department of Thoracic Oncology, King Hussein Cancer Center, P.O. Box 1269, Queen Rania Al Abdullah Street, Amman, 11941 Jordan; 2 Department of Surgery, King Hussein Cancer Center, Amman, Jordan; 3 Department of Radiology, King Hussein Cancer Center, Amman, Jordan; 4 Faculty of Dentistry, Damascus University, Damascus, Syria; 5 Department of Urology, King Hussein Cancer Center, Amman, Jordan; 6 Department of Anesthesia, King Hussein Cancer Center, Amman, Jordan

**Keywords:** Transdiaphragmatic, Single-port VATS, Metastasectomy, Minimally invasive surgery, Case series

## Abstract

**Background:**

Pulmonary metastasectomy was performed in the early twentieth century and ever since, it has evolved to be one of the main treatment options for certain metastatic malignancies. The advancement of minimally invasive procedures enabled new techniques to minimize morbidity and improve patient quality of care and overall outcome.

**Cases presentation:**

Herein we present three patients, aged 53, 48, and 27 years, known to have sigmoid, rectal, and non-seminomatous germ cell tumors respectively. All patients were diagnosed to have metastatic lung nodules and underwent laparotomy to excise abdominal tumors followed by trans-diaphragmatic single-port video-assisted thoracoscopic pulmonary metastasectomy. All patients achieved complete surgical tumor excision, and none had pulmonary related complications on follow-up.

**Conclusion:**

Our prescribed novel trans-diaphragmatic single-port video-assisted thoracoscopic surgery (VATS) technique for synchronous pulmonary metastasectomy and intra-abdominal tumor resection is safe and can achieve complete resection with negative margins.

**Supplementary Information:**

The online version contains supplementary material available at 10.1186/s13019-021-01393-2.

## Introduction

The lungs are the second most frequent site of metastatic growth from extra-thoracic malignancies. A 2-stage synchronous or metachronous surgical approach involving the resection of the metastatic pulmonary nodules and the primary malignancy is the mainstay treatment with a 5-year overall survival of 30–55%. The major hurdle for such option is the extensive dissection involving the abdomen and the thoracic cavity condemning it as over-aggressive [1, 2]. Synchronous intra-abdominal and lung resections provide a favorable outcome in terms of patient quality of life and cost-effectiveness. However, both abdominal and thoracic incisions remain inevitable even with minimally invasive thoracoscopy, leading most surgeons to perform a staged lung resection—i.e., to have patients recover from abdominal surgery prior to lung resection. In an attempt to reduce the invasiveness of simultaneous intra-abdominal tumor resection and lung metastasectomy, we developed a simple and novel technique to resect synchronous lung lesions via a transdiaphragmatic single port video-assisted thoracoscopic surgery (VATS) approach in patients undergoing intra-abdominal resection for metastatic disease. Therefore, when the thoracic cavity is entered from the abdominal cavity via the diaphragm, a thoracic incision can be avoided [3].

## Case presentation

### Patient 1

A 53-year-old non-smoker female with a family history of colon cancer was diagnosed with a stage III sigmoid cancer in 2017. The patient complained of abdominal pain associated with constipation leading to a colonoscopy that revealed a sigmoid adenocarcinoma with a staging whole body Computed Tomography (CT) scan revealing no distant metastasis. She underwent surgical resection followed by adjuvant chemotherapy regimen (Avastin, Oxaliplatin, and Xeloda) of 8 cycles completed on July 2018. Follow-up Positron Emission Tomography (PET)/ (CT) scan showed new left lung lesions for which she underwent left Video-Assisted Thoracoscopic (VATS) metastasectomy, which was converted to thoracotomy on October of the same year.

**Fig. 1:**
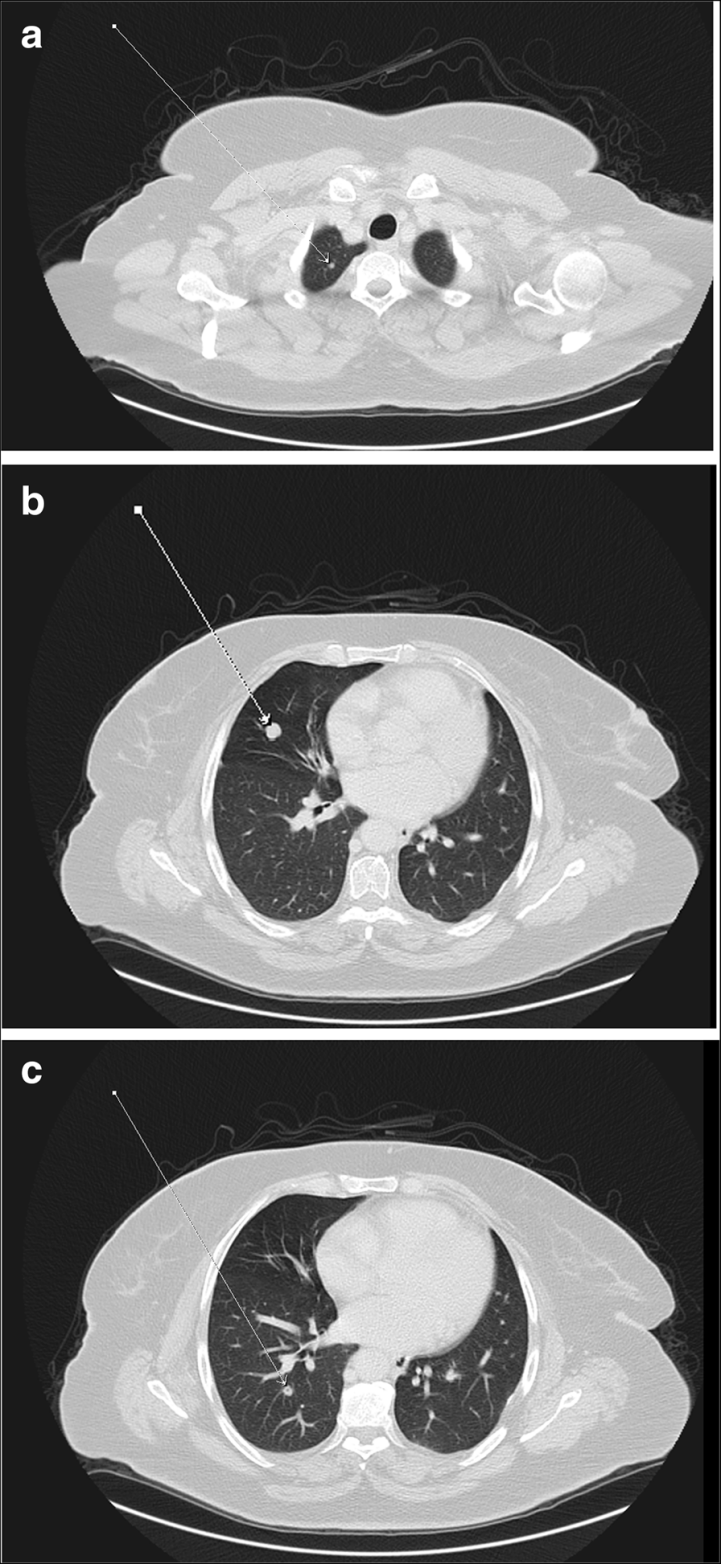
Pre-operative chest CT scan showing the location of three pulmonary nodules in patient 1. Pre-operative chest CT scan – axial view – pulmonary window, showing three right pulmonary nodules located on **a** the superior lobe, **b** lateral part of the middle lobe, and **c** inferior lobe. All indicated by white arrow

**Fig. 2:**
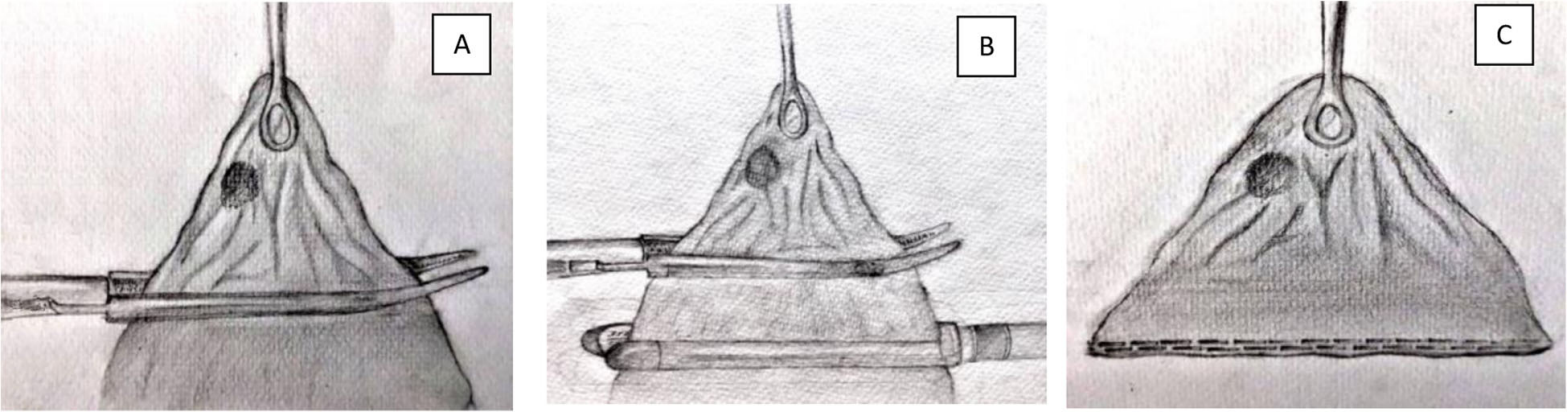
Illustrative figures showing the steps to ensure safe resection margin; **a** Identification of the lung nodule and isolating it using thoracoscopic Roberts clamp while holding it with a thoracoscopic lung clamp. **b** applying the endo-stapler distal to the thoracoscopic Roberts clamp. **c** Complete excision of the lesion with safe margins

On February of 2019, the patient was referred to King Hussein Cancer Center (KHCC) following disease progression with new bilateral pulmonary and liver lesions revealed by a PET/CT scan (the highest SUV max = 4.16 and 15.98 respectively), suggestive of metastatic disease (Fig. 1). A multidisciplinary clinic (MDC) decision after consulting the patient and her family was to go for surgical resection but the patient requested to start a new line of chemotherapy. From July 2019 to November 2019, the patient was started on Irinotecan, and Capecitabine (a total of 6 cycles), and Bevacizumab (a total of 5 cycles). Upon re-evaluation, the metastatic lung and liver lesions remained constant despite adjuvant chemotherapy. On February 2020, the patient was scheduled for surgical resection. Under general anesthesia with the utilization of double-lumen endotracheal tube, midline laparotomy was done followed by right liver wedge metastasectomy; the hepatobiliary surgery team mobilized the right hepatic lobe by opening the right triangular ligament. As the right lung deflated, a 3-cm opening was made in the right diaphragm peripherally to avoid phrenic nerve injury, followed by an installation of dual ring wound protector in the diaphragm opening. With assistance of 30-degree camera and VATS instruments, we mobilized the lung lobe, pushed the diaphragm upward for adequate exposure and palpated the lesions, then we did a wedge resection from upper, middle and lower lobes after we applied a clamp distal to each nodule and cut below the clamp to ensure resection with safety margin using endostapler (Fig. 2). Chest tube was inserted through the 7th intercostal space (ICS) at the anterior axillary line. The lung was inflated, and the diaphragm closed by a continuous-double layer using size 2 Ethibond Excel suture (Ethicon, Inc., Somerville, NJ).

After the procedure, the patient was transferred to the Intermediate Care Unit (IMU) for 24-h under continuous observation. Following that, she was transferred to the surgical ward. The chest tube was removed with a total output of 190 ml of serosanguinous fluid on post-operative day one (POD1). She was followed up during her stay with daily chest x-ray to ensure full lung expansion. The patient was discharged on post-operative day four and histopathological specimen analysis confirmed complete surgical resection (R0) of all excised lesions. The patient was followed up in clinic with no complications.

### Patient 2

**Fig. 3:**
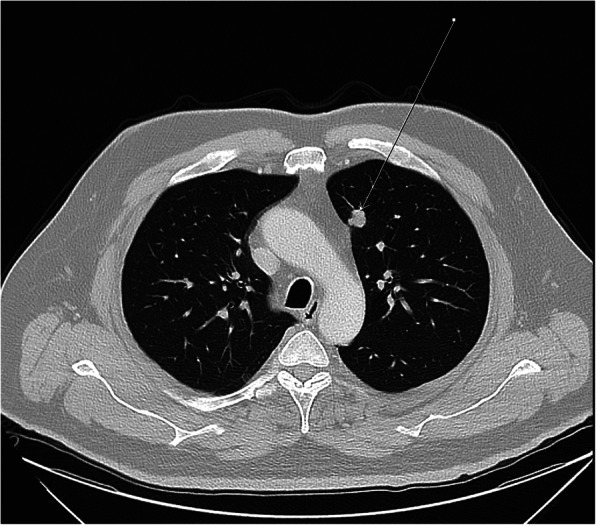
Pre-operative chest CT scan showing the location of pulmonary nodule in patient 2. Preoperative chest CT scan – axial view – pulmonary window, showing a left upper lobe pulmonary nodule as indicated by the white arrow

**Fig. 4:**
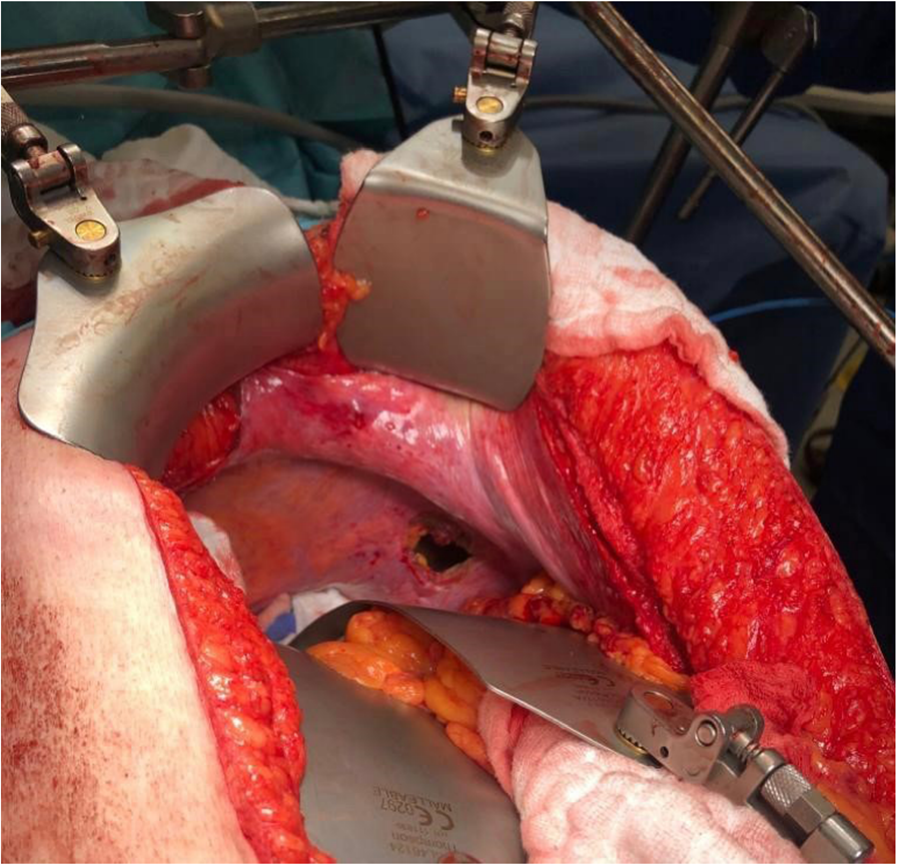
Intra-operative image showing a 3–4 cm incision in the right diaphragm. Intraoperative image showing a 3-cm incision in the left diaphragm after resection of the liver lesions and opening of the left triangular ligament followed by retraction of the left liver lobe inferiorly

**Fig. 5:**
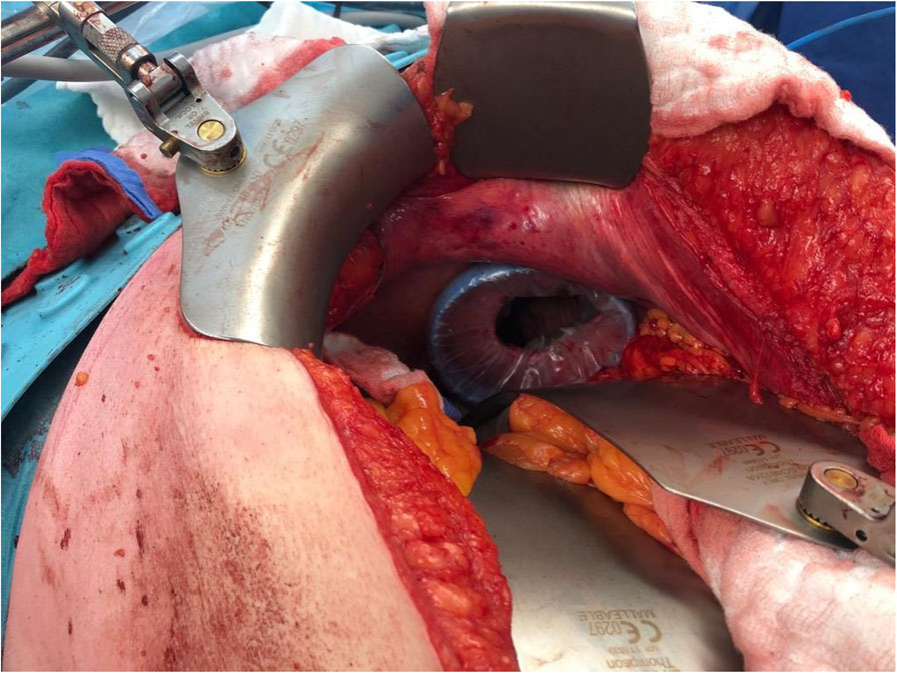
Intra-operative image showing the installation of dual-ring wound protector in the previously made diaphragmatic incision


A 48-year-old male with morbid obesity (Body Mass Index (BMI) of 37.32 kg/m^2^), hypertension (medicated by bisoprolol 5 mg daily) and a strong family history of colon cancer on the paternal side (including his father, brother and aunt) was diagnosed with a stage IV rectal cancer. In January 2019, the patient started to complain of a change in bowel habits associated with bloody stool and a one-month significant weight loss (133 kg to 122 kg). Colonoscopy revealed an ulcerative mass 10 cm from the anal verge and a biopsy confirmed a diagnosis of moderately differentiated adenocarcinoma. The patient was referred to KHCC in April 2019, and a whole-body PET/CT scan showed hypermetabolic circumferential rectal wall thickening with SUVmax = 11.3, few hypermetabolic focal liver lesions in right liver lobe, the most prominent one in segment VIII measuring about 2.6 × 1.7 cm in its active component with SUVmax = 8.12 and hypermetabolic left upper lobe lung nodule measuring about 1.3 cm in its active component with SUVmax = 3.96 (Fig. 3). MDC decision after consulting the patient and his family was to go for 5 cycles of neoadjuvant chemotherapy (irinotecan, leucovorin, 5-fluorouacil) with the last cycle on December 2019. The patient underwent open low anterior resection of the rectal mass with multiple liver wedge resections from segments V, VI, and VIII. Through the midline laparotomy incision, the left triangular ligament was opened to retract the left liver lobe inferiorly. After ensuring left lung deflation via the double-lumen endotracheal tube, a 3-cm opening was made in the left diaphragm through the antero-lateral muscular part (left costal portion of diaphragm) to avoid phrenic nerve injury (Fig. 4) with application of a small-size dual ring wound protector (Fig. 5). Through this single opening, we introduced the 30-degree camera scope and the usual thoracoscopic instruments to perform a pleural adhesiolysis. A 13 mm nodule was identified in the anterior segment of the left upper lobe by pushing the diaphragm caudally while retracting the lung simultaneously. We Applied clamp distal to the nodule and cut below the clamp to ensure resection with safety margin using endostapler (Fig. 2). Chest tube (size 28 F) was inserted through the chest wall at the level of 7th ICS anterior axillary line. Lung inflation was insured, and the diaphragm was closed by a continuous-double layer using size 2 Ethibond Excel suture (Ethicon, Inc., Somerville, NJ) (Video).

**Additional file 1: Video.** Intraoperative video showing the staged approach for lung metastasectomy starting with entering the thoracic cavity, identifying of the lung lesion, isolation and cutting, inserting chest tube, and closure.

The patient was kept under close observation in the IMU for 24 h, followed by transfer to the surgical ward and removal of the chest tube on POD1, which yielded only 10 ml of serosanguinous fluid. He had daily chest x-rays to ensure complete lung inflation and he was discharged on the sixth postoperative day. Definitive histopathologic examination showed completely resected (R0 resection) moderately differentiated adenocarcinoma in the primary tumor site as well as the liver and lung metastasis. The definitive pathological stage was pT3N1 due to involvement of one out of the 13 resected lymph nodes, the patient was placed on adjuvant chemotherapy regimen (irinotecan, leucovorin, and 5-fluorouracil), and had no postoperative complications.

### Patient 3

**Fig. 6:**
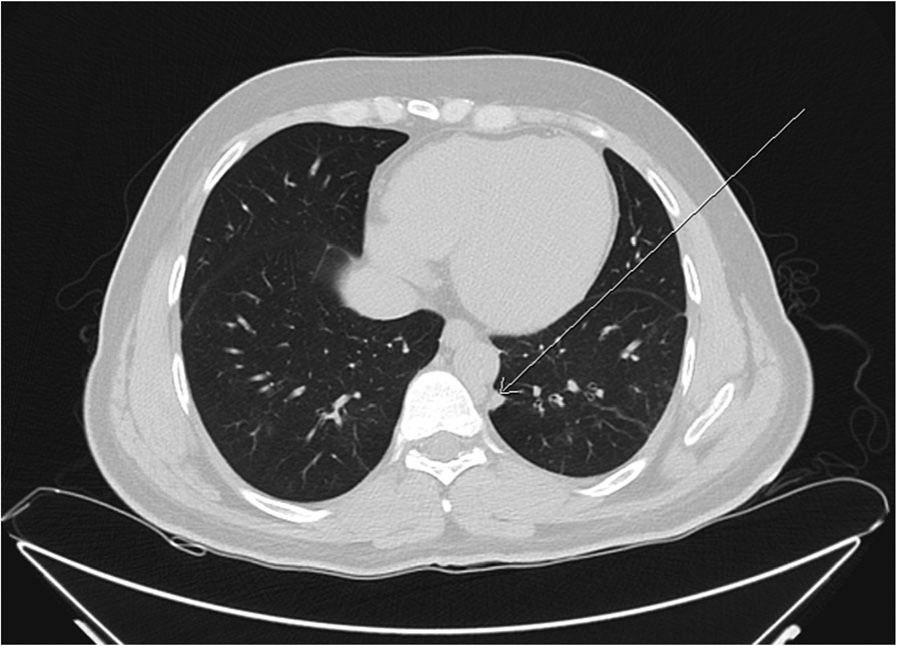
Pre-operative chest CT scan showing the location of pulmonary nodule in patient 3. Preoperative chest CT scan – axial view – pulmonary window, showing a left lower lobe pulmonary nodule as indicated by the white arrow

A 27-year-old male who smokes 30 pack-years and otherwise medically free, started to complain of left progressive testicular swelling in January 2019. Despite being treated surgically as a case of simple hydrocele on July 2019, the swelling persisted necessitating a whole-body CT scan that showed left spermatic cord thickening with abnormal enhancement, enlarged para-aortic lymph nodes, and suspicious pulmonary nodule. Laboratory investigations showed alpha feto-protein (AFP) level of 4.5 ng/mL, beta human chorionic gonadotropin (βHCG) of 135 mIU/ml, and lactate dehydrogenase (LDH) of 248 U/L. He underwent left radical orchiectomy at an outside facility, with a histopathological diagnosis of non-seminomatous germ cell tumor, embryonal type. The patient was referred to KHCC with surgical wound dehiscence, scrotal swelling, pus discharge and necrosis. A new staging CT scan showed multiple bilateral rounded pulmonary nodules suggestive of metastasis with the largest seen at the medial aspect of the left lower lobe measuring about 2.8 cm (Fig. 6), and an irregular left inguinal soft tissue thickening with enhancement and few prominent lymph nodes that may represent postsurgical change. Multiple enlarged metastatic left external iliac, left common iliac and left para-aortic lymph nodes were seen with the largest at left para-aortic region at the level of the left renal hilum measuring about 3.7 cm in short axis with a stage of cT3N2M1aS2. MDC decision after meeting the patient and his family was to start BEP (Bleomycin, Etoposide and Cisplatinum) chemotherapy protocol. He completed four cycles on December 2019, tumor markers normalized, and follow-up imaging studies showed significant regression in the size of the previously noted pulmonary metastasis in the left lung lower lobe to 1.2 cm and decreased size of the previously noted enlarged retroperitoneal and left pelvic lymph nodes. The scan however showed an inferior vena cava (IVC) thrombus of 8 cm in length and the patient underwent thrombectomy using AngioJet Zelante catheter (Boston Scientific, Marlborough, MA) and IVC OptionELITE filter (ARGON Medical Devices, Frisco, TX) insertion. In April 2020, under general anesthesia, the patient underwent retroperitoneal mass excision, bilateral nerve sparing with retroperitoneal lymph node dissection, inguinal scar excision, left spermatic cord excision, left uretrouretorostomy, left double-J insertion, and transdiaphragmatic left single-port VATS lower lobe pulmonary metastasectomy.

Through the midline laparotomy incision, we cut the left triangular ligament to retract the left liver lobe downward. Left lung deflation was successful. It was followed by a 3-cm incision in the left diaphragm through the anterio-lateral muscular part (left costal portion of diaphragm) to avoid phrenic nerve injury and installation of dual ring wound protector. We introduced the 30-degree camera scope and the VATS instruments. We then identified the lower lobe nodule at the posteriobasal segment. We Applied clamp distal to the nodule and cut below the clamp to ensure resection with safety margin using endostapler. Chest tube size 28F was placed and attached to a negative pressure device through the chest wall at the level of 7th ICS anterior axillary line. Lung was inflated and the diaphragm was closed by a continuous-double layer using size 2 Ethibond Excel suture (Ethicon, Inc., Somerville, NJ).

The patient was kept under continuous monitoring for 24 h in the IMU followed by a transfer on POD1 to the surgical ward and removal of the chest tube with an output of zero milliliters. The patient was discharged on post-operative day 6 and was followed up in outpatient clinic without postoperative complications related to lung metastasectomy.

## Discussion

It has been reported that the lung is the second most common site of metastatic focus for almost 25–54% of malignancies developing elsewhere in the body [4] with colorectal, head and neck, and urological tumors having the highest frequencies [5]. Throughout medical history, the emergence of pulmonary metastasectomy has been described as incidental and fortuitous dating back to 1927, when Tudor Edwards described a sublobar resection of a metastatic lower limb sarcoma [6], followed by the first cohort of 25 patients at Memorial Hospital undergoing pulmonary metastasectomy with either a solitary lung lesion or multiple metastases but confined to one lung with an unfortunate 10 out of 25 patients surviving more than 5 years [7].

Pulmonary metastasectomy is performed when; 1) the primary malignancy is controlled or controllable, 2) no extrapulmonary metastasis exists except in colon cancer when lung and liver metastasectomies can be achieved, 3) metastatic nodules are resectable without compromising pulmonary reserve, and 4) medical treatment that has lower morbidity was deemed unsuccessful [8].

The development of minimally invasive thoracic procedures began with Kaiser et al. describing the first VATS operation back in 1995 in suspicious lung lesions [9]. In the year 2000, the da VINCI robot was approved by the American food and drug administration (FDA) for usage in the medical field [10]. Yoshino et al. were the first to utilize the novel technique in thoracic surgery [11]. The first reported case series of laparotomy trans-diaphragmatic approach (LTDA) for a combined procedure was by Dionigi et al. in 2006 using a 5 cm incision around the diaphragmatic hiatus, which was utilized to access the thoracic cavity for pulmonary metastasectomy in two of the three reported cases [12].

Mise et al. published a case series spanning the years of 2000 to 2013 of 16 patients with colorectal cancer and synchronous lung and liver metastases who underwent LTDA via bilateral or right subcostal, inverted L, J-shaped, or diaphragmatic midline incisions. Authors did not state the length of the incisions made nor the timing of chest tube removal. He indicated that such an approach can be done on peripheral lung nodules, with a distance of less than 3 cm from the pleural, regardless of lung lobe [3]. A 10 cm diaphragmatic incision around the central tendon was reported by Lerut et al. to access the thoracic cavity in the process of liver and lung metastasectomies [13].

Twenty-eight patients who underwent laparoscopic transdiaphragmatic VATS procedures using two-port entry sites to access the thoracic cavity with curative intent due to various etiologies, were reported by Andrade et al. Authors did not report an exclusion criterion but recommended avoiding such procedure in patients with a body mass index (BMI) ≥ 35 kg/m2. In addition, they recommended avoiding the left transdiaphragmatic approach in relatively young patients or those who practice heavy lifting due to increased possibility of herniation [14].

A reverse trans-thoracic approach via thoracotomy incision, with intra-abdominal liver segment resection was described by Delis et al. Authors reported 5 patients with metastatic colorectal cancer, two of which developed pleural effusion [15]. Possible contraindications for this approach include but are not limited to tenacious adhesions in the pleural space; inability to obtain or to tolerate mono-pulmonary ventilation; pulmonary hilar lesions; neoplastic infiltration of the thoracic wall and contaminated or infected abdominal entity [12].

All studies reported chest tube insertion before wound closure without emphasizing how long it remained inside the chest cavity and when it was removed. They also reported pulmonary complications, such as pneumonia, air-leak, atelectasis, and pleural effusion without a clear postoperative timeline [3, 14, 15].

In order to avoid phrenic nerve injury and possible diaphragmatic paralysis, an incision should start anteriorly just lateral to the pericardium extending circumferentially as far posteriorly as needed, keeping in mind that incision through the central tendon will provide only minimal exposure with a low chance of diaphragmatic paralysis. In case other types of incisions were to be attempted, meticulous care should be carried to avoid the anterolateral and posterolateral branches of the phrenic nerve.

**Fig. 7:**
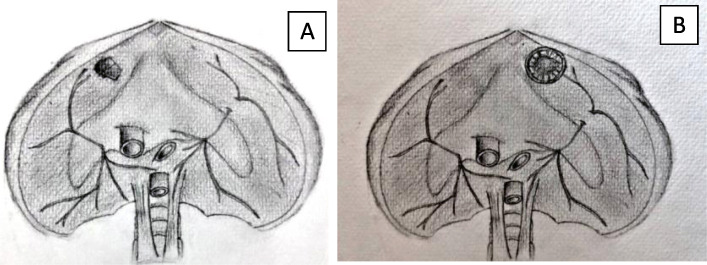
Illustrative figures showing **a** the location of the diaphragmatic incision ensuring phrenic nerve safety and **b** showing an installation of the dual ring wound protector

In the cases reported in this series, we entered the thoracic cavity via a 3–4 cm incision in the antero-lateral muscular part of the diaphragm (Fig. 7), avoiding the phrenic nerve and its branches. A limitation of the transdiaphragmatic approach is its narrow visual field in the thoracic cavity; we overcame this obstacle by using the single port VATS technique for better visualization, which permitted the safe performance of adhesiolysis in one of our cases. We utilized pre-operative chest CT scan for localization of the anatomical locations of the nodules. Following lung lesion detection and identification, we placed a curved clamp distal to the lesion and then applied an endostapler to cut below the clamp to ensure negative resection margin (Fig. 2).

Based on meticulous literature review of reported procedural details, no description of dual ring wound protector usage on the diaphragm opening to facilitate the introduction of the VATS instruments and to protect the diaphragm wound from being contaminated by the tumor was identified. In addition, this case series is the first to report chest tube removal on the first post-operative day. Even though we accessed the thoracic cavity via a small single incision, we were able to achieve resection with negative margins in all of the three reported cases. Based on the Sniff test performed on patient two and three, after 6 and 3 months post-operatively respectively, no abnormality in diaphragm function was detected.

**Fig. 8:**
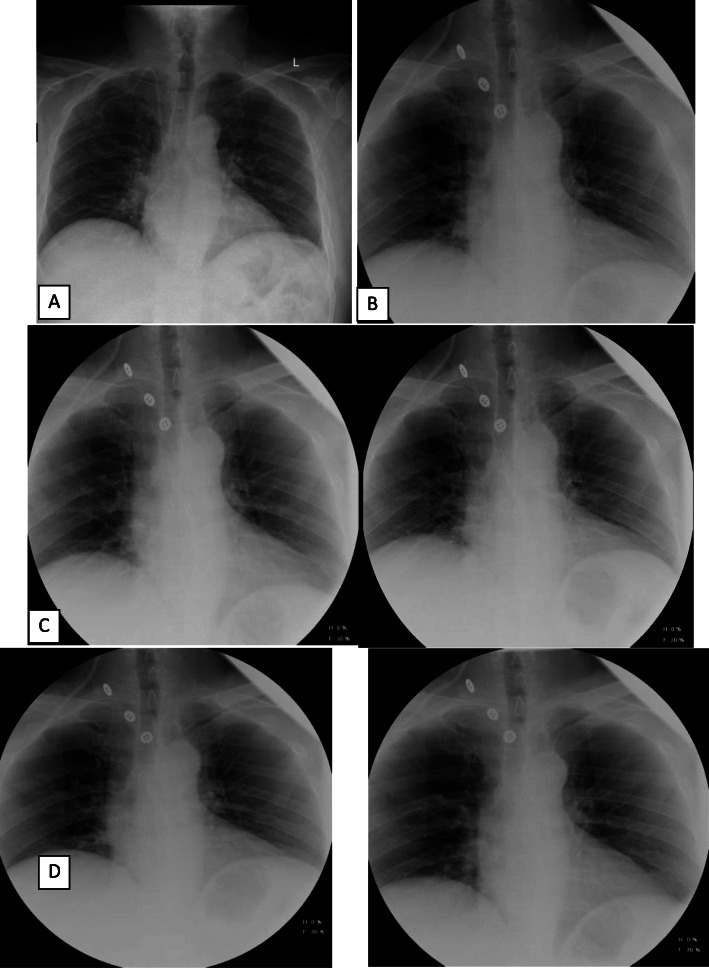
Post-operative postero-anterior chest sniff test x-rays. Posteroanterior chest X-rays, **a** Preoperative posteroanterior radiograph showing normal height of both hemidiaphragms. **b** Postoperative posteroanterior radiograph showing normal height of both hemidiaphragms. **c** On quiet breathing, normal orthograde motion of both hemidiaphragms. **d** On deep respiration, normal orthograde motion of both hemidiaphragms. No delayed or paradoxical movement

For postoperative follow up, we can assess the diaphragm for any dysfunction using diaphragmatic sonography. This method is considered the preferred modality in children and young adult [16]. Dynamic magnetic resonance has been described but is not widely adopted [17]. The optimal test by far is the fluoroscopic sniff test where functional radiographs are used to assess the diaphragmatic motion during inspiration and expiration (Fig. 8). In normal conditions, both hemidiaphragms should move downward, but in case of paralysis in one hemidiaphragms, paradoxical motion is seen. This is exaggerated to bilateral paradoxical movements when both hemidiaphragms are paralyzed and reduced to only a delay in movement in case of weakness [18].

## Conclusion

Simultaneous transdiaphragmatic resection of lung lesions in patients undergoing intra-abdominal tumor resection is safe. Minimal invasive VATS utilizing a 3 cm single port access through the diaphragm to the chest cavity is a novel approach that can allow a complete R0 pulmonary metastasectomy with no surgical complications and diaphragm function was spared, shown by post-operative functional assessment with sniff test, as seen in our case series. This low-invasive technique will facilitate aggressive surgical treatment for synchronous intra-abdominal tumor and lung metastases.

## Data Availability

Not applicable.

## Abbreviations

VATS: Video-assisted thoracoscopic surgery
PET: Positron emission tomography
CT: Computed tomography
KHCC: King Hussein Cancer Center
MDC: Multidisciplinary clinic
ICS: Intercostal space
POD1: Postoperative day 1
BMI: Body mass index
AFP: Alpha-feto protein
βhCG: Beta human chorionic gonadotropin
LDH: Lactate dehydrogenase
IVC: Inferior vena cave
